# Research on Evaluation of University Emergency Management Ability Based on BP Neural Network

**DOI:** 10.3390/ijerph20053970

**Published:** 2023-02-23

**Authors:** Ruili Hu, Ye Zhang, Longkang Wang

**Affiliations:** 1 Department of Security, University of International Business and Economics, Beijing 100029, China; 2 School of Foreign Studies, University of International Business and Economics, Beijing 100029, China; 3 China Center for Information Industry Development, Beijing 100048, China

**Keywords:** colleges and universities, emergencies, emergency management ability, evaluation indicators, BP neural network

## Abstract

University emergency management ability is an important part of university safety management. To evaluate university emergency management ability scientifically, objectively, and accurately, this study constructs three first-level indexes, namely, pre-prevention ability, in-process control ability, and post-recovery ability, and 15 s-level indexes, including the establishment of emergency management institutions; the construction of emergency plans; the allocation of emergency personnel, equipment, and materials; and the training and exercise of emergency plans. On the basis of the backpropagation (BP) neural network method and MATLAB platform, an evaluation model of university emergency management ability is constructed. The neural network evaluation model is trained with sample data, and a university in Beijing is adopted as an example to verify the good prediction effect of the model. The results show that applying the evaluation model based on the BP neural network to the emergency management ability of colleges and universities is feasible. The model provides a new method to evaluate the emergency management ability of colleges and universities.

## 1. Introduction

At present, the security and stability situations of colleges and universities are generally stable and good, but against the background of the intertwined and overlapping changes over the last century and the ongoing pandemic, many unstable and unsecure factors emerge, and security challenges are intensifying. Poisoning and killing, laboratory explosion, food poisoning, campus violence, student suicide, and other emergencies occur from time to time and show an increasing trend. Their frequent occurrence poses great challenges to the safety management of colleges and universities and the harmony and stability of society. In the face of emergencies in colleges and universities, China has established the emergency management system of planning and legislative/institutional/regulatory systems (known in China as “One Planning Plus Three Systems”) [1]. As early as 2007, China has formulated and promulgated a series of laws and regulations, such as the “Emergency Response Law” [2], that provide institutional guidance and methods for emergency handling in colleges and universities. Colleges and universities have also issued policy documents on campus public safety emergency management, but college emergencies are characterized by high uncertainty, unpredictability, subject specificity, and public proliferation, which have imposed strict requirements on the emergency management capacity of universities and colleges. Therefore, conducting research on the evaluation of the emergency management capacity of universities and colleges is important.

In foreign countries, the earliest research on the management of university emergencies was conducted in developed countries, such as the United States and Japan. The Federal Emergency Management Agency and the National Emergency Management Association jointly investigated and launched a self-assessment tool (Capacity Assessment for Readiness, CAR) [3] for assessing the response capacity of each region to emergencies. CAR is a comprehensive evaluation index system containing 13 primary indicators, 209 secondary indicators, and 1014 tertiary indicators [4]. Foreign scholars have also conducted exploratory research on emergency response capacity assessment. For example, Fink [5] proposed the four-stage life cycle model, which has laid a systematic theoretical foundation for academic research on the management of university emergencies. Daniel Weisdorf [6] explained the importance and status of emergency response capacity assessment and emphasized that the indicators of emergency response capacity should be in a dynamic process. American scholars have posited that the core point of emergency response lies in prevention. In addition, the U.S. Federal Education Agency has summarized the evolution of emergency events in colleges and universities into four stages: mitigation and prevention, preparation, response, and recovery. Universities around the world establish their emergency response plans based on the four processes. Meanwhile, Japanese scholars have conducted extensive research on the management of university emergencies [7]. The Japanese scholar Wakai Yaichi [8] pointed out that the management of university emergencies includes several aspects, such as health care, school accident liability, environmental management, safety education, and management.

Domestic scholars have also conducted extensive research. Weike Chen et al. [9] adopted Hall’s 3D structure and established an evaluation index system for the public emergency management capability of colleges and universities. Jianxin Yu et al. [10] established a backpropagation (BP) neural network evaluation model for campus security in colleges and universities. Qinghua Liu et al. [11] combined rough set and gray correlation analysis methods to perform a comprehensive evaluation of the emergency management capability of colleges and universities. Hao Ji et al. [12] constructed an evaluation index system of the emergency management capability of universities that considers the risk factors affecting such capability; they used fuzzy hierarchical analysis to construct an evaluation model of the emergency management of universities. Xu Zhao [13] built an evaluation model for the emergency management capability of universities by using improved grey hierarchical analysis. Based on the theories of crisis life cycle and comprehensive emergency management, Rui Sun [14] constructed an evaluation index system of the emergency response capability of universities that covers three primary indicators (i.e., prevention capability, control capability, and recovery capability) and 15 secondary indicators (including emergency plan design, safety system inspection, plan training, and drill) and evaluated the emergency response capability based on a fuzzy mathematical model. Jiale Hao [15] constructed an emergency management system for universities from a system perspective and modeled and quantitatively analyzed the system by using a Petri net. Kang Sun et al. [16] compared and analyzed the application of the entropy method, principal component projection, combined optimization model of entropy method, and principal component projection in the evaluation of the public emergency management capability of universities. Yang Xu et al. [17] constructed a grading evaluation model of public crisis warning in universities from the two dimensions of crisis events and crisis management and proposed an improved evaluation method of alpha-intercept fuzzy TOPSIS.

Most of the above-mentioned studies have used hierarchical analysis and fuzzy comprehensive evaluation to establish different evaluation systems or models, which provide a theoretical basis for the evaluation of the emergency management capability of universities. However, these methods cannot easily eliminate the subjectivity and arbitrariness of the evaluators in determining the index weights, which affect the evaluation results. The BP neural network overcomes the subjectivity of evaluation indexes in the process of assigning weights and has achieved excellent results in network security, quality security, project security, security management, and ecological security evaluations [18,19,20,21,22]. At present, only a few studies have applied BP neural networks to the evaluation of the emergency management capacity of universities. On this basis, this study adopts the BP neural network method to design and establish an evaluation model of the emergency management capacity of universities. The accuracy and reliability of the evaluation method and model are verified through an example application. Thus, it can better provide a new method and idea for the evaluation of emergency management ability in colleges and universities.

## 2. Construction of an Evaluation Index System

The establishment of an evaluation index is a crucial step in the process of safety evaluation. The rationality of index formulation directly affects the accuracy of evaluation results. By combing and analyzing a large amount of domestic and foreign related literature, we initially selected index sources suitable for the evaluation of the emergency response capacity of Chinese universities. Then, we combined the actual situation of Chinese universities and the actual situation of a Beijing university, which is the empirical research object of this study, and designed an index system of campus emergency management capability with a certain degree of reliability and validity.

The evaluation index system contains 1 target level, 3 first-level indexes, and 15 s-level indexes. The first-level indexes are pre-prevention ability, in-process control ability, and post-recovery ability. Specifically, pre-prevention ability includes the establishment of emergency management institutions (B_11_); the construction of emergency plans (B_12_); the allocation of emergency personnel, equipment, and materials (B_13_); the training and exercise of emergency plans (B_14_); and detection, identification, and early warning capabilities for emergencies (B_15_), for a total of five second-level indexes. In-process control ability includes the counter speed of emergency handling plan (B_21_); the activation and implementation of emergency plans (B_22_); the dissemination, collection, processing, and transmission of information (B_23_); on-site organization and command ability (B_24_); emergency coordination ability (B_25_); and equipment device and technology (B_26_), for a total of six second-level indexes. Post-recovery ability includes the accountability mechanism (B_31_), accident investigation (B_32_), recovery and reconstruction capability (B_33_), and psychological crisis prevention and counseling capability (B_34_), for a total of four second-level indexes. The specific indexes are shown in Table 1.

## 3. BP Neural Network Methods and Principles

### 3.1. BP Neural Network Model

**Figure 1:**
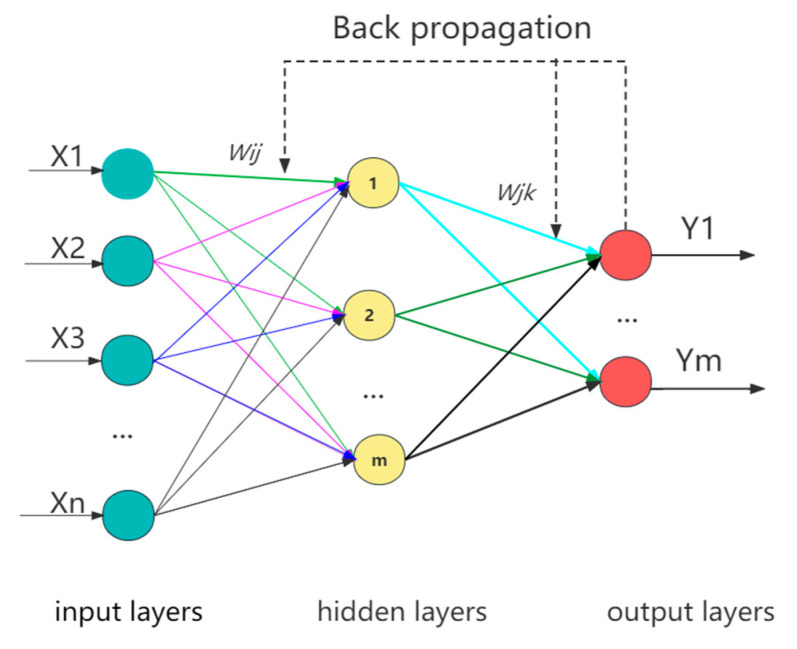
BP neural network topology.

BP is a multilayer forward feedback network trained by data forward propagation and error back propagation invented by a research group led by Rumelhart and McClelland in the United States in 1986; it masters a large number of input–output transfer mappings and can store and remember these mappings [23]. The BP neural network structure is composed of an input layer, a hidden layer, and an output layer, each of which consists of a number of nodes (also called nerves). Compared with other evaluation methods, BP neural network has the following advantages: (1) the creation, training, and use of neural network can be completed by MATLAB to realize data evaluation and analysis; (2) it has a strong logic processing ability, and a good nonlinear mapping relationship exists between input and output data [24]; and (3) it has sufficient fault tolerance, and the partial damage of neurons in each layer does not affect the operation results of the whole network [25]. Its structure is sketched in Figure 1.

### 3.2. Algorithm Principle of BP Neural Networks

The BP neural network operation process includes two parts. The signal goes from the input layer to the output layer through the hidden layer, indicating a forward calculation process. The error is calculated by comparing the output signal with the expected output value. When the error is greater than the specified range, the output result is back-propagated and the weights and thresholds between layers are adjusted one by one according to the error value. The two computation processes are repeated until the resulting error is within the given accuracy range; then, the neural network training is completed [26]. The specific algorithm flow is as follows.

**Figure 2:**
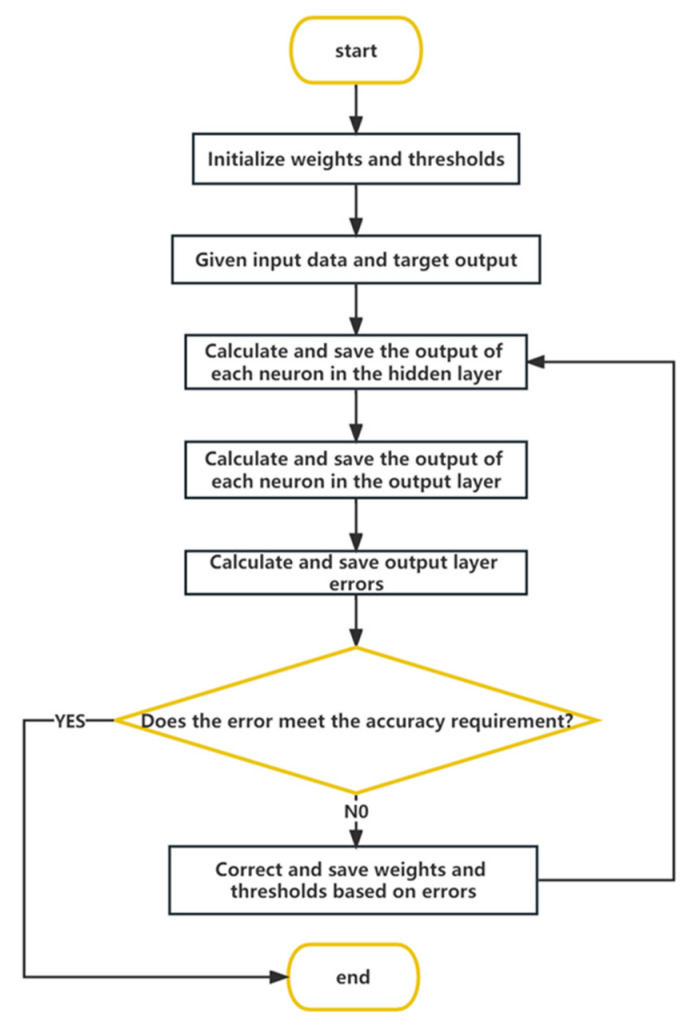
Flow chart of the BP neural network algorithm.

(1) Sample selection and pre-processing. In accordance with the research objectives, suitable training and test samples are selected. The sample data are normalized because of the different activation functions of BP neural networks.
(2) Network initialization. In accordance with the nature of the input and desired output values of the trained data, the total number of input layer neurons (s), the total number of hidden layer neurons (r), and the number of output layer neurons (t) of the training network are decided. The training accuracy, number of iterations, neuron excitation function, and training function are also set.
(3) Operation of the output value of the implicit layer. Sample data *p* are inputted into the layer, and the output value *F* of the hidden layer is calculated with Equation (1), where *w_ij_* is the connection weight between the input layer and the hidden layer, *a* is the threshold value of the neurons in the hidden layer, *f* is the excitation function of the neurons in the hidden layer, and *r* denotes the number of neurons in the hidden layer.
  (1) $$F_{j}=f\left[\sum_{i=1}^{s}w_{ij}p_{i}-a_{j}\right],\;j=1,\;2,\;3,\;\ldots ,\;r$$
  Many kinds of excitation functions can be used for BP neural networks. The network excitation function selected in this study is the hyperbolic tangent logsig function with the following expression:
  (2) f(x)=1/(1+e^(−x)).
(4) Calculation of the output value of the output layer. The output value *Y* of the output layer is calculated with Equation (2), where f is the output value of the implicit layer of the network, *w_jk_* is the connection weight between the implicit layer and the output layer, and *b* is the threshold value of the output layer.
  (3) $$Y_{K}=\sum_{j=1}^{r}F_{j}w_{jk}-b_{k},\;k=1,\;2,\;\ldots ,\;t$$
(5) Neural network forward propagation error calculation. The expected output value already available is *Z*. The error value of network prediction is calculated with Equation (4), where *Y* is the network output value calculated by the forward propagation of the neural network.
  (4) $$V_{k}=Z_{k}-Y_{k},\;k=1,\;2,\;\ldots ,\;t$$
(6) Update of connection weights between layers. New connection weights are calculated from the network prediction error values, and the new connection weights are *W_ij_* and *W_jk_*. The specific formula is as follows:
  (5) $$W_{ij}=w_{ij}+\eta F_{j}\left(1-F_{j}\right)p\left(i\right)\sum_{k=1}^{t}w_{jk}V_{k},\;i=1,\;2,\;\ldots ,\;s;\;j=1,\;2,\;\ldots ,\;r,\;$$
  (6) $$W_{jk}=w_{jk}+\eta F_{j}V_{k},j=1,\;2,\;\ldots ,\;s;\;k=1,\;2,\;\ldots ,\;t,\;$$
  where *η* is the learning efficiency.
(7) Update of the thresholds for each layer of backpropagation. The new thresholds are *C_j_* and *D_k_* with the following equations:
  (7) $$C_{j}=c_{j}+\eta F_{j}\left(1-F_{j}\right)\sum_{k=1}^{t}w_{jk}V_{k},\;j=1,\;2,\;\ldots ,\;r,\;$$
  (8) $$D_{k}=d_{k}+V_{k},\;k=1,\;2,\;\ldots ,\;t.\;$$
(8) In accordance with the expected set error range for analysis, whether the output value meets the accuracy requirements is determined; when it does, the operation ends, and the result is outputted. Otherwise, the network iteration continues, and Step (3) is implemented again to continue the training calculation until the error accuracy requirements are met. The calculation process of BP is shown in Figure 2.

## 4. Construction of the Evaluation Model

### 4.1. Training Sample Acquisition

In this study, five universities in City B were selected as the data source for the training sample, and 10 experienced experts from emergency management government departments, emergency management professional colleges and universities, and emergency management scientific research institutions were invited to use the expert scoring method to quantify and score the capability values of each index of the five universities. The scoring was based on a 100-point system, and each index was scored in the range of 0–100 from poor to excellent. The emergency response capability of universities was classified into five levels, with reference to Table 2. The level corresponding to the emergency response capability of universities was determined based on the target value, namely, the emergency response capability score, outputted from the neural network. The scores given by experts are shown in Appendix A.

### 4.2. BP Neural Network Design

Research has already confirmed that a three-layer neural network can meet the needs of model building and data operation [27], so a three-layer feedforward neural network model was established in this study. The number of input neurons of the BP neural network was determined using the previously established evaluation index of the emergency management capability of universities, and the input layer was represented by X (X = 15). The number of neurons in the hidden layer is directly related to the training accuracy of the network, so we determined the number of neurons in the hidden layer by adopting the empirical formula

(9) $$Y=\sqrt{M+N}+b$$

where *Y* represents the number of neurons in the hidden layer, *M* is the number of identified nodes in the input layer, *N* is the number of identified nodes in the output layer, and *b* is a positive integer that takes values from 1 to 10.

On the basis of experience, the number of neurons in the hidden layer is generally set as an integer in the range of 5–14. After repeated verification in this study, the number of neurons in the hidden layer was designed to be 10. The final evaluation result was only 1, that is, the number of neurons in the output layer was set to 1. Thus, a BP neural network with the structure of “15-10-1” was used as the research evaluation model.

### 4.3. BP Neural Network Model Building and Training

The MATLAB platform was adopted as a basis for BP model building and training in this study. First, the data were normalized using the mapminmax function. The hyperbolic tangent logsig function was selected for modeling, the linear function purelin was adopted as the activation function, traingda was used as the training function, and the BP network creation function newff was employed to establish the BP network structure. The fitting number, network training number, learning efficiency of network training, and error accuracy of network training were set to 20, 9999, 0.1, and 0.001, respectively.

**Figure 3:**
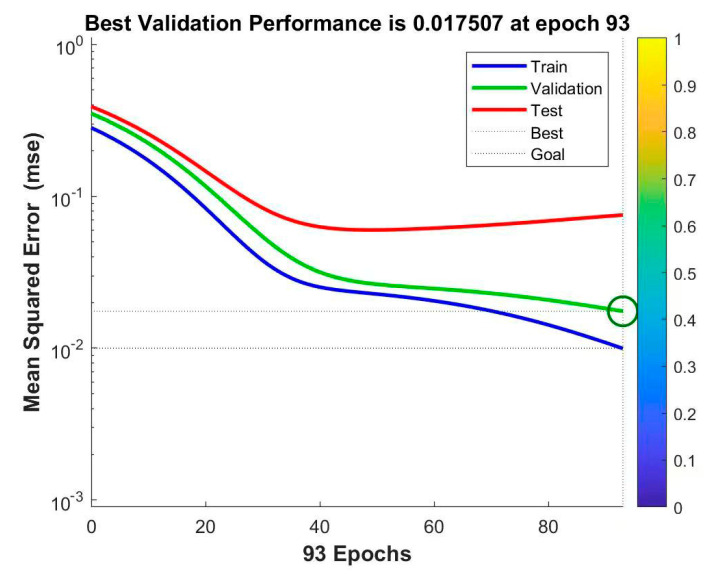
Output iterative precision curve.

**Figure 4:**
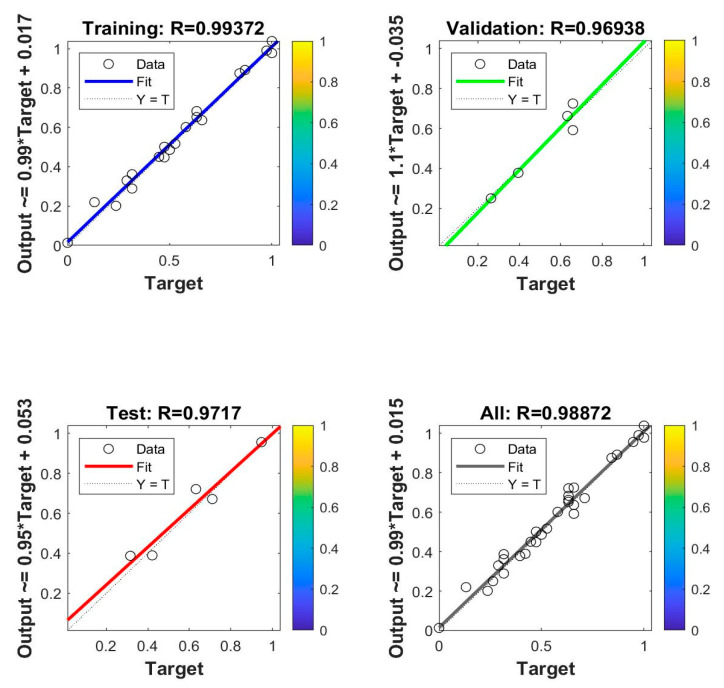
Training, validation, and test results.

The existing data were divided into training, validation, and test data. The training data were involved in training the prediction model, and the validation and test data were used to validate and test the accuracy of the model, respectively. The training of the whole network was stopped when any of the following three conditions were met: the fitting training did not converge for 20 consecutive times, the maximum number of iterations set was reached, and the training accuracy was less than or equal to 0.001. At the end of the iteration, network training was terminated, and the iterative accuracy curve (Figure 3) and the training, validation, and testing results (Figure 4) were outputted.

As shown in Figure 3, the training was completed after the 103rd iteration, and the training termination accuracy was 0.00965, which was lower than the expected accuracy target set at this time. This result indicates that the model training fitting effect of this BP neural network met the accuracy setting requirements.

The data dispersion distribution in Figure 4 shows the model’s fitting effect. In the model of regression prediction, R2 is generally used to judge the accuracy of the model, and the value of R2 in the model is between 0 and 1. When R2 is less than 0.5, the prediction of this model is problematic. When R2 is equal to 0.6, the model can explain 60% of the prediction result. When R2 is greater than 0.75, the prediction result of the model is good. According to Figure 4, R = 0.99372 and R2 = 0.9875. R2 is greater than 0.75, indicating that the accuracy of the model is high.

To compare the BP neural network training results with the expected output results, the model operation was performed using the an = sim (net, inputb_test) function. The prediction results were outputted by the inverse normalization process of the mapminmax (reverse) function, and the relative error was calculated by comparing the prediction results with the true values. The results are shown in Table 3.

**Figure 5:**
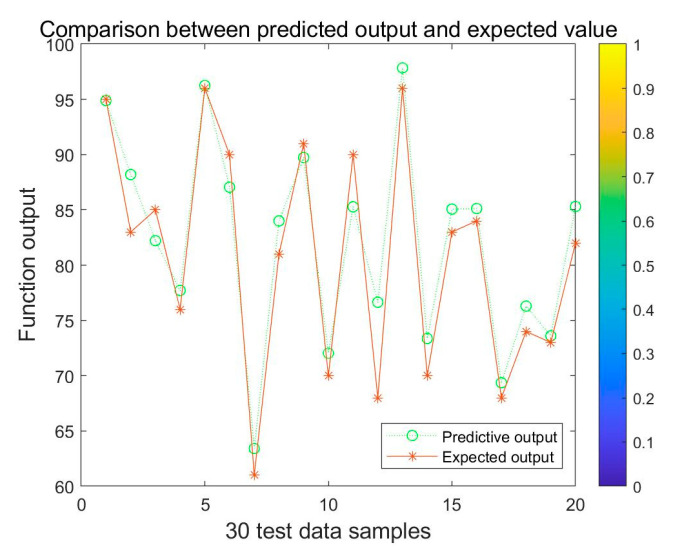
Comparison of training and expected output results.

The training results are plotted against the desired output in Figure 5.

It can be seen from Figure 5 that the virtual line of predicted output value in green almost coincides with the real line of actual expected value in red, and the error rate is relatively low, indicating that the simulation and prediction ability of the model is relatively strong. Meanwhile, as shown in Table 3 and Figure 5, the training results of the BP neural network conformed to the expected output results with small errors, and the coefficient of determination of the model was as high as 0.96999, indicating that the training results of the BP neural network model were accurate and had higher prediction accuracy. Hence, the model can be used feasibly for the evaluation of the emergency management capability of universities.

Furthermore, the mean absolute percentage error (MAPE) was adopted for model validation.

(10) $$MAPE=\frac{100\%}{n}\sum_{i=1}^{n}\left|\frac{{\hat{y}}_{i}-y_{i}}{yi}\right|,$$

where *n* is the sample size of the data set, *y_i_* is the measured value of the *i*-th sample, and ${\hat{y}}_{i}$ is the predicted value of the BP neural network for the *i*-th sample.

The average absolute percentage error of this BP neural network model in the test set of the emergency management capability of colleges and universities was 0.02953. According to the error analysis, this BP neural network model can be used to conduct an evaluation study of university emergency management ability effectively and reasonably.

## 5. Example Application

With a university in Beijing as an example, we invited 10 experts in related fields to score the evaluation indexes of university emergency management ability in accordance with the actual situation of the university. The scores of the experts are shown in Table 4, where Z denotes the experts.

The scoring data of the 10 experts on the emergency management capability of a university in Beijing (Table 4) were imported into MATLAB workspace, and the name of the prediction data was input_ forecast data by using the trained BP neural network. The prediction was implemented as follows:

% normalize the predicted data

inputb_ forecast data = mapminmax(‘apply’, input_forecast data, inputps);

%predicted data is substituted into the trained BP neural network model

An = sim(net, inputb_ forecast data);

% denormalize the predicted data

Routput = mapminmax(‘reverse’, an, outputps);

% shows the predicted results

Result = Routput

Result = 82.3419 83.2315 83.4819 83.9694 82.2223 81.0435 83.5739 84.3361 85.4231 86.6456

The 10 evaluation results were close to one another. According to the scoring rules, the evaluation result of the emergency management ability of this university is excellent, which is consistent with the overall judgment and recognition of the plan by experts and related personnel.

In general, the neural network model for the evaluation of university emergency management ability can evaluate the level of emergency management accurately and can provide a management basis for university managers.

## 6. Conclusions

As social education institutions, colleges and universities have social importance. The occurrence of university emergency events affects the normal teaching and living order of colleges and universities and produces unacceptable repercussions in society, which are not conducive to the construction of a harmonious campus and society. Strengthening the management of university emergencies, establishing an ideal emergency mechanism, and improving the ability of universities in dealing with emergencies can promote the safety and stability of campus environments. Therefore, performing an accurate, scientific assessment of university emergency management ability is essential. The emergency management ability of higher-education institutions involves many departments and complicated elements and is difficult to assess. Most of these elements are measured qualitatively. Through the analysis of relevant literature, the current study developed a scientific evaluation index system that uses BP neural networks with the aid of the MATLAB platform. Evaluation models were constructed, trained, and applied to transform the qualitative evaluation into a highly intuitive quantitative evaluation.

This research is summarized as follows:

By sorting and generalizing a large amount of related literature, we constructed an index system for evaluating university emergency management ability. The system consists of three first-level indexes and 15 s-level indexes. The first-level indexes are pre-prevention ability, in-process control ability, and post-recovery ability. The 15 s-level indexes include the establishment of emergency management institutions (B_11_); the construction of emergency plans (B_12_); the allocation of emergency personnel, equipment, and materials (B_13_); and the training and exercise of emergency plans (B_14_). On the whole, the index system is scientifically rich and covers many elements of university emergencies.

At present, the methods for emergency management evaluation in colleges and universities mostly adopt the hierarchical analysis method and the fuzzy synthesis method with subjective weight color. Many factors affect emergencies in colleges and universities, which are complex systems, and these factors are difficult to quantify. The BP neural network method has excellent self-learning and nonlinear processing abilities, and the weights in the calculation process can be automatically adjusted in accordance with the fitting error, thereby minimizing human subjectivity. Thus, the BP neural network method helps improve the scientific evaluation of university emergency management ability.

In this study, five universities in City B were selected as data sources of training samples, and 10 experts in related fields were invited to quantify and score the ability values of each index of the five universities. Using the BP neural network with the help of the MATLAB platform, a BP neural network model was constructed. The BP neural network model was checked and tested by training with the experts’ quantitative scoring data. The comparison of the results revealed that the BP neural network evaluation model can be applied effectively in the evaluation of university emergency management ability and can achieve a scientific assessment of such ability.

On the basis of the BP neural network model, we evaluated the emergency management ability of a university in Beijing. The evaluation result of the emergency management ability of the university was excellent and in line with the actual situation. Thus, the evaluation model has good applicability.

This paper focuses on the application of the BP neural network in the evaluation of university emergency management ability. However, there are still things in the paper that need to be explored further. On the one hand, there are many factors affecting university emergencies, and each school has its own characteristics. Therefore, more targeted and applicability indexes should be selected in the evaluation of different universities. On the other hand, we can continue to increase the selection of sample size to obtain higher evaluation accuracy.

## Figures and Tables

**Table 1:** Index system of campus emergency management capability.

First-Level Index	Second-Level Index	Index Description
Pre-prevention ability B_1_	Establishment of emergency management institutions B_11_	Whether the university sets up emergency management institutions and whether the settings are reasonable.
	Construction of emergency plans B_12_	Whether the emergency plan is set up according to the university’s own situation and whether the emergency response plan for emergencies is complete.
	Allocation of emergency personnel, equipment, and materials B_13_	The structure and number of personnel at each level of the department in dealing with emergencies and the availability of emergency equipment and supplies (includes whether the equipment is complete and whether the supplies are sufficient).
	Training and exercise of emergency plans B_14_	Whether training and rehearsal of emergency plans for colleges and universities are conducted and whether the rehearsal is reasonable.
	Detection, identification, and early warning capabilities for emergencies B_15_	The ability to detect emergencies, the ability to analyze and identify the development trend of possible emergencies, and the ability to use relevant information websites to obtain relevant information.
In-process control ability B_2_	Counter speed of emergency handling plan B_21_	The speed of activation of the plan after the occurrence of a contingency.
	Activation and implementation of emergency plans B_22_	After the occurrence of an emergency, whether to start and implement the plan in accordance with the principle of graded response and whether the implementation of the plan is in place.
	Dissemination, collection, processing, and transmission of information B_23_	Whether the information release is timely, whether the information collection is comprehensive and true, and whether the information transmission is effective.
	On-site organization and command ability B_24_	Whether the command staff configuration is reasonable to deal with emergencies and whether the command, control, and coordination mechanisms are sound.
	Emergency coordination ability B_25_	After the occurrence of an emergency, whether the communication and collaboration between relevant personnel are smooth and close, respectively.
	Equipment device and technology B_26_	After the occurrence of an emergency, whether the equipment and devices meet the needs and whether the rescue technology is mature.
Post-recovery ability B_3_	Accountability mechanism B_31_	Whether an accountability mechanism exists and whether the rewards and punishments are reasonable.
	Accident investigation B_32_	Whether the cause of the accident and the situation of responsibility for the accident are investigated and analyzed, whether information materials related to the accident are collected, and whether the situation is reported to the higher authorities (government investigation team).
	Recovery and reconstruction capability B_33_	Whether the recovery and reconstruction are timely after the occurrence of emergency events.
	Psychological crisis prevention and counseling capability B_34_	After the occurrence of an emergency, whether psychological counseling is provided to relevant personnel, whether the manner is correct, and whether the effect is significant.

**Table 2:** Scoring levels and corresponding values.

Level	Level	Score
I	Excellent	[100,80)
II	Good	[80,60)
III	Fair	[60,40)
IV	Poor	[40,20)
V	Very poor	[20,0)

**Table 3:** Error table of test result data and expected result data.

Data Serial Number	Expected Result	Test Result	Error Value
1	72	71.8215	0.1785
2	65	64.5552	0.4448
3	72	72.7988	−0.7988
4	79	79.0697	−0.0697
5	79	78.9891	0.0109
6	73	73.7639	−0.7639
7	90	89.9139	0.0861
8	78	77.3807	0.6193
9	78	77.4545	0.5455
10	85	84.9267	0.0733
11	87	87.6468	−0.6468
12	74	73.6516	0.3484
13	78	78.7856	−0.7856
14	68	67.5989	0.4011
15	62	61.8829	0.1171
16	85	84.8470	0.153
17	69	69.6545	−0.6545
18	68	67.6010	0.399
19	67	66.4145	0.5855
20	96	96.0437	−0.0437

**Table 4:** University emergency management ability quantitative score table.

Index	Z_1_	Z_2_	Z_3_	Z_4_	Z_5_	Z_6_	Z_7_	Z_8_	Z_9_	Z_10_
B_11_	85	83	84	89	85	90	88	85	87	84
B_12_	79	84	81	80	75	80	75	80	85	82
B_13_	70	72	79	75	68	80	74	73	77	80
B_14_	60	61	59	57	55	69	60	58	60	55
B_15_	75	80	86	75	72	75	70	73	81	72
B_21_	50	55	58	55	56	60	59	55	53	56
B_22_	75	80	72	73	85	80	88	78	90	78
B_23_	70	65	60	68	75	62	60	60	65	64
B_24_	70	76	56	78	90	60	80	70	80	78
B_25_	80	85	87	88	83	85	90	87	85	86
B_26_	85	87	85	80	81	85	80	75	83	85
B_31_	90	95	93	90	90	92	94	93	95	92
B_32_	80	86	85	81	80	83	80	80	84	84
B_33_	90	91	90	95	90	88	92	85	89	90
B_34_	89	90	87	67	50	87	90	92	98	78

## Data Availability

The data that support the findings of this study are available from the corresponding author upon reasonable request.

## Appendix A

**Table A1:** BP neural network model training sample data.

Index	B_11_	B_12_	B_13_	B_14_	B_15_	B_21_	B_22_	B_23_	B_24_	B_25_	B_26_	B_31_	B_32_	B_33_	B_34_	M
S_1_	85	76	92	65	70	76	92	90	86	73	96	95	87	85	74	81
S_1_	90	90	90	80	85	90	80	85	85	80	90	80	85	80	80	85
S_1_	100	95	98	95	100	99	96	95	89	98	97	92	93	96	91	95
S_1_	80	70	80	70	60	50	70	70	80	60	70	70	60	70	70	67
S_1_	80	70	79	78	70	78	78	78	70	70	70	70	70	70	70	73
S_1_	80	60	70	50	40	60	50	70	90	50	60	80	50	50	40	63
S_1_	80	75	80	91	82	76	72	86	75	80	80	85	84	75	82	83
S_1_	90	90	100	98	90	90	92	90	90	90	90	95	100	100	100	96
S_1_	90	80	80	90	90	90	90	90	60	90	90	90	90	90	80	84
S_1_	80	60	90	80	50	65	60	60	60	60	70	80	70	90	90	73
S_2_	70	60	80	50	60	30	60	70	80	70	70	70	70	90	90	70
S_2_	80	78	75	76	77	65	72	80	80	80	75	66	78	79	85	76
S_2_	80	75	60	80	70	60	70	80	60	60	60	60	60	75	75	68
S_2_	60	60	70	50	60	60	60	60	50	50	60	60	70	80	80	63
S_2_	90	98	100	98	100	55	91	95	90	92	91	95	90	89	92	90
S_2_	60	70	80	60	70	100	80	70	60	50	60	60	60	70	60	68
S_2_	70	50	70	70	40	60	90	80	70	50	60	60	70	90	85	69
S_2_	70	80	66	45	60	80	70	70	60	40	50	70	70	80	70	67
S_2_	98	100	95	96	90	100	95	99	100	100	100	100	96	100	100	98
S_2_	80	70	75	90	75	90	85	85	75	80	85	75	70	80	75	81
S_3_	75	80	80	50	75	90	75	80	80	90	85	90	90	90	85	82
S_3_	70	60	80	20	60	80	80	80	70	60	60	80	80	80	80	70
S_3_	100	80	80	90	70	60	80	80	80	80	90	70	80	69	78	79
S_3_	80	70	80	80	80	80	90	90	95	98	90	75	70	90	80	82
S_3_	85	96	56	78	45	89	76	85	98	87	78	96	96	98	75	83
S_3_	95	95	96	20	95	96	97	97	95	95	96	96	96	96	95	90
S_3_	50	60	80	30	40	60	80	80	50	50	60	60	80	70	80	61
S_3_	85	70	75	80	75	80	80	86	85	84	80	85	80	89	86	80
S_3_	65	75	75	100	80	75	74	70	72	71	76	80	85	86	86	77
S_3_	90	80	90	95	92	90	95	90	90	80	90	95	95	95	95	91

In the table, B_mn_ refers to the 15 evaluation indexes, S_K_ denotes the school number, and M is the overall value of dormitory safety management evaluation.
